# Is ^18^F-FDG PET/CT a real breakthrough imaging test in predicting the outcome of percutaneous ablation of metastases?

**DOI:** 10.1590/0100-3984.2019.52.2e1

**Published:** 2019

**Authors:** Carlos Alberto Buchpiguel

**Affiliations:** 1 Full Professor in the Department of Radiology and Oncology of the Faculdade de Medicina da Universidade de São Paulo (FMUSP), São Paulo, SP, Brazil. Email: buch@usp.br.

Percutaneous ablation has been extensively investigated as an alternative treatment for patients with indolent tumors or tumors for which surgical resection is not feasible. Despite its growing acceptance among oncologists and surgeons, many questions remain regarding the best technique (e.g., radiofrequency, cryoablation, or microwave ablation) and how this therapy affects overall survival, especially in patients with liver metastasis due to colorectal cancer^(^^1^^)^. The COLLISION trial, an ongoing phase III, single-blind prospective randomized controlled trial, will probable answer some of these questions^(^^2^^)^. Nevertheless, the reported accuracy of percutaneous ablation is 80-85%^(^^2^^)^.

In cases of lung cancer, percutaneous ablation has been advocated as a highly efficient alternative therapy for highly selected patients with stage IA tumors or with oligometastatic lung disease, mainly when surgical resection is not possible because of severe clinical comorbidities or potential complications related to the surgical approach^(^^3^^)^. In a recent prospective multicenter trial, percutaneous ablation showed a success rate of 85% in stage IA non-small cell lung cancer, in patients who are not candidates for surgery^(^^4^^)^.

Positron emission tomography/computed tomography (PET/CT) has emerged as a molecular imaging tool that detects disease more on the basis of the molecular profile or metabolic cellular signaling than on structural or functional abnormalities. The high rate of anaerobic glycolysis is one of the main features of various malignant tumors, and that is the reason for using ^18^F-fluorodeoxyglucose (FDG), a common positron emitter produced in a cyclotron^(^^5^^)^. In addition to its well-validated application in staging, monitoring treatment responses, and detecting tumor recurrence, the role of ^18^F-FDG PET/CT in predicting the effectiveness of percutaneous ablation is still under discussion and investigation.

A very interesting paper published in the previous issue of **Radiologia Brasileira**, authored by Romanato et al.^(^^6^^)^, represents the first study enrolling patients that were referred for ^18^F-FDG PET/CT performed immediately after percutaneous ablation (_iPA_^18^F-FDG PET/CT). The authors showed a good correlation between the _iPA_^18^F-FDG PET/CT findings and those of the follow-up imaging studies. Albeit a relative expensive tool for local or segmental evaluation, _iPA_^18^F-FDG PET/CT had a false-positive rate that was low (7.6%), although it was still higher than that reported in patients referred for PET/CT performed at a time point that was slightly longer after ablation^(^^7^^)^. As very well discussed by Romanato et al.^(^^6^^)^, that difference might be partially explained by the fact that a lung tumor treated with cryoablation and liver metastasis from colorectal cancer treated with radiofrequency ablation were included in the same group for analysis. The relatively low sensitivity in detecting a viable tumor is dependent not only on the amount of viable cells still present after ablation but also on many other biological features that might be impaired immediately after ablation, such as the enzymatic activity of hexokinase, proliferation activity of the tumor cells (in the vicinity and within the target), and hypoxia-inducible factors in the periphery of the treated lesion. Performing the PET control study as early as possible, in order to increase the chance that inflammatory cells will be present around the treated region, might also help guide the interventional radiologist decision to complement the ablation if the _iPA_^18^F-FDG PET/CT shows that focal areas of residual increased metabolism persist. That might be the most practical insight from the article for promising future applications of _iPA_^18^F-FDG PET/CT in percutaneous ablation. However, in order to answer the question of whether PET could really be a better method of guiding the ablation therapy, it will be necessary to conduct a prospective, randomized single-blind study and compare the rates of complete ablation, and perhaps the clinical outcomes, between the two groups. Another aspect that would be very interesting to investigate is the value of _iPA_^18^F-FDG PET/CT in improving overall survival and recurrence-free survival rates in a highly selected group of patients, in comparison with that of the standard approach (performing only imaging studies during a follow-up period of the standard duration). Although we still cannot answer the question raised by this editorial, the Romanato et al.^(^^6^^)^ article provided very interesting insights into the use of ^18^F-FDG PET/CT after percutaneous ablation of malignant cells in a selected group of patients.
